# On the Challenge of Interpreting Census Data: Insights from a Study of an Endangered Pinniped

**DOI:** 10.1371/journal.pone.0154588

**Published:** 2016-05-05

**Authors:** Fritz Trillmich, Kristine Meise, Stephanie Kalberer, Birte Mueller, Paolo Piedrahita, Ulrich Pörschmann, Jochen B. W. Wolf, Oliver Krüger

**Affiliations:** 1 Animal Behaviour, University of Bielefeld, Bielefeld, Germany; 2 Department of Evolution, Ecology and Behaviour, Institute of Integrative Biology, University of Liverpool, Biosciences Building, Liverpool, United Kingdom; 3 Center for Biology Education, University of Münster, Münster, Germany; 4 Facultad de Ciencias de la Vida, Escuela Superior Politécnica del Litoral, ESPOL, Guayaquil, Ecuador; 5 Huinay Scientific Field Station, Huinay, Los Lagos, Chile; 6 Dept. of Evolutionary Biology, Uppsala University, Uppsala, Sweden; Institute of Ecology, GERMANY

## Abstract

Population monitoring is vital for conservation and management. However, simple counts of animals can be misleading and this problem is exacerbated in seals (pinnipeds) where individuals spend much time foraging away from colonies. We analyzed a 13-year-series of census data of Galapagos sea lions (*Zalophus wollebaeki*) from the colony of Caamaño, an islet in the center of the Galapagos archipelago where a large proportion of animals was individually marked. Based on regular resighting efforts during the cold, reproductive (cold-R; August to January) and the warm, non-reproductive (warm-nR; February to May) season, we document changes in numbers for different sex and age classes. During the cold-R season the number of adults increased as the number of newborn pups increased. Numbers were larger in the morning and evening than around mid-day and not significantly influenced by tide levels. More adults frequented the colony during the warm-nR season than the cold-R season. Raw counts suggested a decline in numbers over the 13 years, but Lincoln-Petersen (LP-) estimates (assuming a closed population) did not support that conclusion. Raw counts and LP estimates were not significantly correlated, demonstrating the overwhelming importance of variability in attendance patterns of individuals. The probability of observing a given adult in the colony varied between 16% (mean for cold-R season) and 23% (warm-nR season) and may be much less for independent 2 to 4 year olds. Dependent juveniles (up to the age of about 2 years) are observed much more frequently ashore (35% during the cold-R and 50% during the warm-nR seasons). Simple counts underestimate real population size by a factor of 4–6 and may lead to erroneous conclusions about trends in population size.

## Introduction

Information about the abundance of animals is essential to understand population processes and it provides an indispensable baseline for conservation and management efforts. However, most population assessments produce relative numbers or indices of population abundance, leaving the absolute numbers uncertain. This can be problematic as relative numbers or indices of abundance may be strongly influenced, for example, by processes of habitat choice: if optimal habitats–which researchers often select for their studies–are chosen by more dominant animals, but are filled up by individuals from less adequate habitats when a population declines (the so-called buffer effect [1–4]), this leads to a shift in distribution as predicted by ideal free distribution models [5,6]. As a consequence, population declines may be overlooked or noticed too late, because the effect can be minimal in optimal habitats. Whereas approximation to an ideal-free distribution may normally be achieved by dispersal of young animals (or first breeders), in periods of major changes in population size–for example, due to drastic shifts in food abundance, prey distribution or high disease mortality—dispersal may additionally involve previously well established, site-faithful older individuals [7] distorting the assessment of population abundance.

In addition to such shifts in population distribution, density-dependent (competition, predation, spread of diseases) and density-independent factors (climate variability, like El Niño events [8]) are expected to influence population dynamics [9]. Moreover, given intrinsic variability in time series of count data [10] and potential threshold effects of changes in food abundance or accessibility, long time series may sometimes be required to detect even drastic changes in population abundance [10–13]. Detecting changes in population size is further complicated by autocorrelation in datasets, as expected in time series of long-lived animals such as large mammals [14]. For example, if recruitment fails, a change in adult numbers may only be noticed with a delay of several years.

In species making long foraging excursions from a central place, population assessment is further complicated. This is well illustrated in seals that spend a major part of their life away from the colony foraging at sea and may visit alternative haul-out sites to rest [15,16]. Therefore, any census at a given site will always only represent an unknown proportion of the total population and this proportion is likely to differ among age classes and sexes. In addition, changes in foraging behaviour induced by shifts in prey distribution may also affect the probability of encountering an animal ashore. Therefore, mark-recapture techniques are needed to properly assess population size [17].

Galapagos sea lions (*Zalophus wollebaeki*) are non-migratory. Because of their limited distribution and apparent decline over the last 30–40 years, they have recently been classified as “Endangered” [18]. Although their year-round presence in the colonies and high site fidelity [19] facilitate the estimation of population size in principle, few solid data are available on population size and trends in numbers [20–22]. This is partly explained by the finding that numbers counted in a given colony fluctuate widely among individual censuses [21]. To date, it is only known with certainty that numbers are strongly affected by El Niño events [23–25].

To understand which factors influence the probability of observing individuals of different ages and status on land and to define a potential sampling scheme for future population monitoring, we analyzed a 13-year-series of censuses from our study colony on Caamaño, in the center of the Galapagos archipelago. Based on intense efforts to resight marked individuals every year during the reproductive, cold season and for many years during the non-reproductive, warm season, we (i) document changes in numbers across the reproductive and non-reproductive season for different sex and age classes, (ii) estimate the trend in population size over the 13 year period of our study, and (iii) use tagging and bleach markings to derive Lincoln-Petersen estimates of the total number of adults and immatures in the colony. In addition, we analyse how different covariates like sea surface temperature (SST), time of day, and tide level influence the numbers of animals encountered ashore.

## Methods

Permission for this study was granted by the Servicio Parque Nacional Galapagos, Ecuador. We studied the sea lion colony on Caamaño, a small islet about 2 km in front of Puerto Ayora, Santa Cruz, in the center of the Galapagos archipelago (0°45‘ S, 90° 16’ W). The islet is about 300 m in diameter [26]. Our study covers the years 2003 to 2015. Overall, we conducted 389 counts across the entire island (Table 1), with 82 counts in 10 warm, non-reproductive (warm-nR) seasons (presence on the island between February and May) and 307 counts during 13 cold, reproductive (cold-R) seasons (presence between September and January) (S1 Table). Counting of animals was done by walking around the island, also covering the inner sections during the survey. For the year 2006, detailed census data by category exist only for three censuses and for 2005, our observations cover less than half (essentially only October) of the cold-R season. In the particularly short warm-nR seasons of 2009 and 2014 we obtained only 2 counts, each.

**Table 1:** Number of total counts in the years 2003 to 2015.

Year	Season	Period (day.month)	Number of counts	mean interval between counts [days]
2003	warm-nR	12.3.-27.4.	36	1.3
2003	cold-R	17.9.-19.11.	31	2.1
2004	cold-R	8.9.-21.11.	28	2.7
2005	cold-R	1.10.-7.11.	15	2.6
2006	cold-R	18.9.-26.11.	24*	3.0
2007	warm-nR	6.3.-27.3.	8	3.0
2007	cold-R	13.9.-14.1.2008	42	3.0
2008	warm-nR	3.3.-16.3.	5	3.0
2008	cold-R	15.9.-20.1.2009	43	3.0
2009	warm-nR	28.4.-1.5.	2	3.0
2009	cold-R	11.9.-6.12.	18	5.0
2010	warm-nR	13.4.-25.4.	4	4.0
2010	cold-R	28.9.-9.12.	25	3.0
2011	warm-nR	5.3.-24.3.	7	3.2
2011	cold-R	8.9.-7.12.	17	5.6
2012	warm-nR	9.3.-12.4.	6	6.8
2012	cold-R	20.9.-5.12.	14	5.8
2013	warm-nR	26.2.-12.3.	6	2.8
2013	cold-R	23.9.-8.12.	14	5.8
2014	warm-nR	22.3.-27.3.	2	5
2014	cold-R	7.10.-30.11.	14	4.2
2015	warm-nR	5.3.-19.3.	6	2.8
2015	cold-R	3.10.-14.12.	22	3.4
**Total**	**warm-nR**	**26.2.-1.5.**	**82**	
**Total**	**cold-nR**	**8.9.-20.1.**	**307**	

Counts in the years 2007 and 2008 include a few censuses in January 2008 (n = 5) and January 2009 (n = 7), respectively. Period refers to the dates (day and month) between the first and last census in a given season. Cold-R = cold, reproductive season; warm-nR = warm, non-reproductive season

*In 2006 only 3 counts with data according to animal categories

With the exception of the warm-nR season of 2003, census intervals were usually spaced 3–6 days apart (Table 1). A census took about 45 min to 1 hour and was done by 3 or 4 trained observers, who simultaneously counted subsections of the islet. The subdivision of the islet into subsectors, marked by clearly visible stakes at 20 m intervals around the island perimeter, made double counting highly unlikely. Every animal encountered was noted and we recorded for every animal if it was marked. We distinguished the following categories: pups, immatures, adults (large individual, sex unknown), adult male and adult female. Pups are recognizable by their unmoulted fur which fades from a blackish brown at birth to light brown shortly before moult at around 5–6 months. As many pups have moulted before the warm season, we did not distinguish between pups and immatures in the warm season censuses and jointly refer to these categories as “juveniles”. Average adult females weigh about 70 kg and measure 156 cm [27] and animals clearly smaller than this, but no longer in pup fur were categorized as immatures. Based on comparison with known-age tagged individuals, immatures comprise animals between 1 and 4(-5) years of age. Adult males are generally larger than adult females [27]. However, males of about female size can also be distinguished from females by their stronger, broader head and wider front flippers. Categorization uncertainties mostly concern the immature category [28]. However, since most animals of this category are the 1 and 2 year-olds, which are clearly smaller than adult females (and almost all of which are tagged), this problem is unlikely to cause major bias. All observers were thoroughly trained at the beginning of each season to identify these categories until we achieved more than 90% agreement in classification.

In addition, regular observation rounds covering the whole island were also performed at least 3 times daily to monitor the presence of marked animals. Animals were marked by double-tagging in the trailing edge of the front flippers (Allflex® sheep ear tags; see [26] for details of capture and tagging procedures) or, in the case of males which were too large for capture with hand nets, approached while resting and given individually recognizable bleach marks (using Wella Blondor® 12%, lasting for 4–6 months; see [29,30] for details).

**Fig 1:**
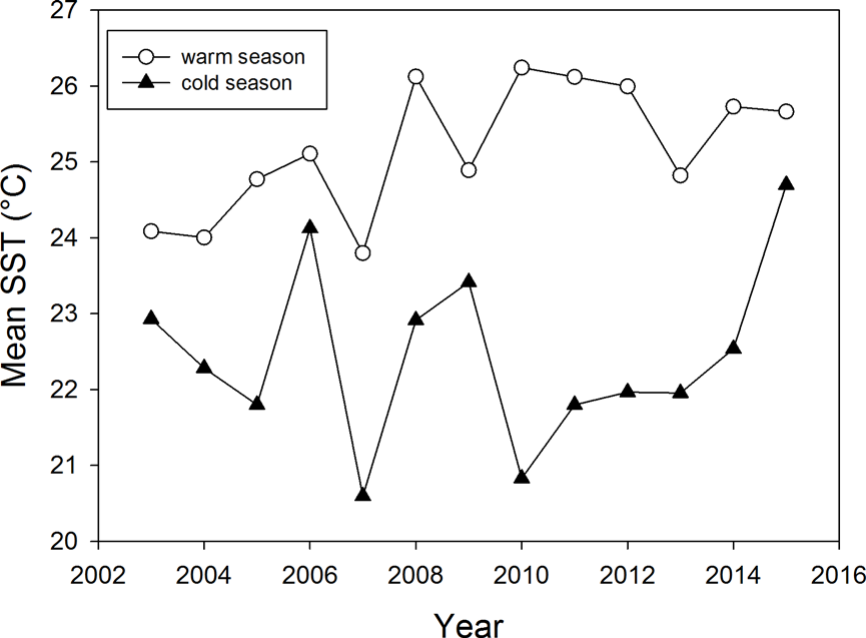
Means 2003–2015 of sea surface temperatures (SST) for the cold, reproductive (September to December; black triangles) and the warm, non-reproductive (February to May; open circles) seasons.

For every census, we noted time of day and determined the tide levels from the tide table for Isla Baltra (http://tides.mobilegeographics.com/locations/2806.html), about 34 km north of Caamaño. We calculated the tide level at the beginning of our censuses by linearly extrapolating between high and low tide level as recorded in the tide table for the respective date. Daily measurements of sea surface temperature (SST) were provided by the Charles Darwin Foundation (http://www.darwinfoundation.org/datazone/climate/). To test for effects of marine conditions, we calculated mean SST for 10 days before and including the day on which the census took place (referred to as SST_10_). Additionally, we calculated the mean SST for each season by averaging SSTs over the period from September to December for the cold-R and for January to April for the warm-nR season, respectively. These are referred to as SST_c_ and SST_w_, respectively, and are shown for the study years in Fig 1.

Year was used as a random variable in those linear mixed effects models (LME; R function *lmer* from the package *lme4*) calculated to extract effects which were general for all years. For modelling changes in numbers ashore within a season using linear models (LM, R function *lm*), we determined the influence of tide level, time of day (between 05:30 and 18:00; categorized as “morning” (begin of census between 5:30 and 8:59), “mid-day” (9:00 to 14:59) and “afternoon” (15:00 to 18:30) and SST_10_ on the numbers of animals counted ashore (S1 Table). As the number of counts varied between years, we repeated the analysis (using linear models; LM, R function *lm*) and tested the influence of these factors separately for each of the cold-R seasons of the years 2003–2005 and 2007–2015 (Table 1). We did the same analysis for the warm-nR seasons of 2003, 2007, 2008, 2011, 2012, 2013 and 2015, years for which we have 5 or more censuses within one warm-nR season (Table 1). Clearly, the analyses for the warm-nR seasons have less power than those for the cold-R seasons, because of the relatively reduced number of censuses per parameter.

We subsequently evaluated changes in overall numbers across the 13 years for the cold-R (when we were present between September and December/January) and the warm-nR seasons (present between February and early May). To determine trends in census size of the population on Caamaño, we modeled the total number of animals (without pups) counted in a given census as well as the different age classes and sexes separately as a function of “year” (2003–2015; using year as a continuous variable), day of the year (1–365; called ‘date effect’) and SST_C_ or SST_W_ during the respective season using linear models. To avoid an undue influence of the 2003 seasons (see Table 1), for that year we used only data from censuses 3 days apart.

To determine the number of animals in the colony, we calculated Lincoln-Petersen (LP) estimates (N = Mn/m), where *N* is the number to be estimated, *M* the number of marked animals in the population, *n* the number counted in a given census and *m* the number of marked individuals encountered in that census. This estimate was calculated for adults and immatures/juveniles separately (S2 Table). As the best estimate of the number of marked animals (*M*) in the population, we determined the number of all tagged adult animals plus individually identifiable bleached animals seen at least twice within a season (to exclude tag reading errors or bleaches that did not take). For immatures, we also used single observations of tagged individuals, since many of the 3 and 4 year-old animals are known to visit the colony only rarely and therefore may be observed only once during a given season. This age category comprises weaned but not yet mature individuals. Since nearly all juveniles were marked, the estimated number of marked juvenile animals quite closely reflects the total number of these animals in the colony. Animals marked within the current season were included in the total number of marked animals only one day after marking.

In 2003, when we initiated our tagging program, we took the number of animals marked until one day before the census as the estimate of animals marked. However, given that animals spend much of their time offshore, this may violate the assumption of perfect mixing between marked and unmarked animals. Similarly, the data obtained for the cold season of the year 2005 differ substantially from those obtained in the other years, as our census activity covered a much narrower period in 2005 and we may therefore not have observed all marked animals using the colony (see Table 1).

We believe the simple Lincoln-Petersen estimate for closed populations to be most appropriate [31]. It makes the following assumptions: 1) The probability of capturing (here resighting) an individual is the same for all individuals in the population. 2) No animal is born or immigrates into the study area between mark and recapture. 3) Marked and unmarked animals die or leave the area at the same rate, and (4) no marks are lost. With regard to assumption 1): We can quite closely approach (within 1–2 m) and, if marked, identify all individuals in our population, so that this assumption appears to be met. 2) Within a season, only pups are born and we record this in detail. Immigration seems negligible. This is evidenced by the almost complete lack of observations of our marked animals from any of the surrounding colonies (Plazas and Santa Fé, both about 30 km away), where tourist guides regularly visit and would have reported to us, had they seen marked animals. This apparent lack of emigration of our animals to other nearby islands implies that immigration to Caamaño is also minimal. This seems to be true for the immatures and juveniles, given that our LP estimates are so close to the actual numbers of tagged animals (see below and also [32]). Indeed, in the later years of the study, we had essentially all juveniles marked, at least late in the season. We know that adult females and males show very high site fidelity [19,29]. More precisely, the latter authors suggest that 82% of all males that have survived to two years of age return regularly as adults to their natal colony. However, we also know that during their foraging sojourns, adult females haul out at places other than their home colony [15,16]. This could lead to the inclusion of animals from other colonies in our censuses and might introduce an error, which we cannot account for. 3) We have no reason to assume that marked and unmarked animals should die or leave the area differentially. Moreover, the probability of death within a season of a given year is low. 4) Tag loss within a given season is negligible.

Using Chapman’s bias-reduced modification of the Lincoln-Petersen index (S2 Table) for calculating adult population size yielded estimates differing only by 8.1 ± 4.2 adult animals (for the cold season; out of about 570 adults estimated) and did not change any of our conclusions.

Linear and linear mixed effects models were developed in R 3.1.2 [33] using the *lm*- and *lmer* functions. Models were calculated first taking all variables and their interactions into account and were subsequently simplified stepwise by removing non-significant interactions and variables. Linear models with additive effects provided an equally good fit as models taking into account interactions (ANOVA comparing models, n.s.). Therefore, we consider only the additive effects. We selected the best models using model comparison with the F-test or with likelihood tests. Model selection using AIC yielded qualitatively the same results.

## Results

### Factors influencing numbers of animals seen ashore

Initially, we tested the influence of environmental factors on the number of animals counted **within** a given season always taking “Year” into account as a random factor. We first report the results for the cold, reproductive (cold-R) and thereafter for the warm, non-reproductive (warm-nR) season.

#### Cold-R season

**Fig 2:**
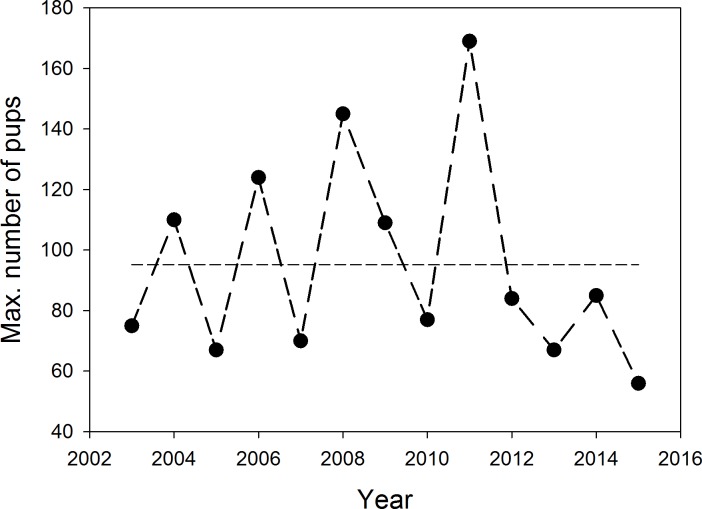
Total number of pups born in the study years as determined by marking of all newborn individuals. This number is greater than the numbers counted as shown in Fig 3B, since many pups die within the first month after birth and a single census rarely, if ever, records all pups present. Hatched line: 13-year mean of number of pups born. No recognizable overall trend exists in the numbers of pups born (linear regression; R^2^ = 0.0207; p = 0.639), even though pup numbers dropped over the last five years (2011–2015).

**Fig 3:**
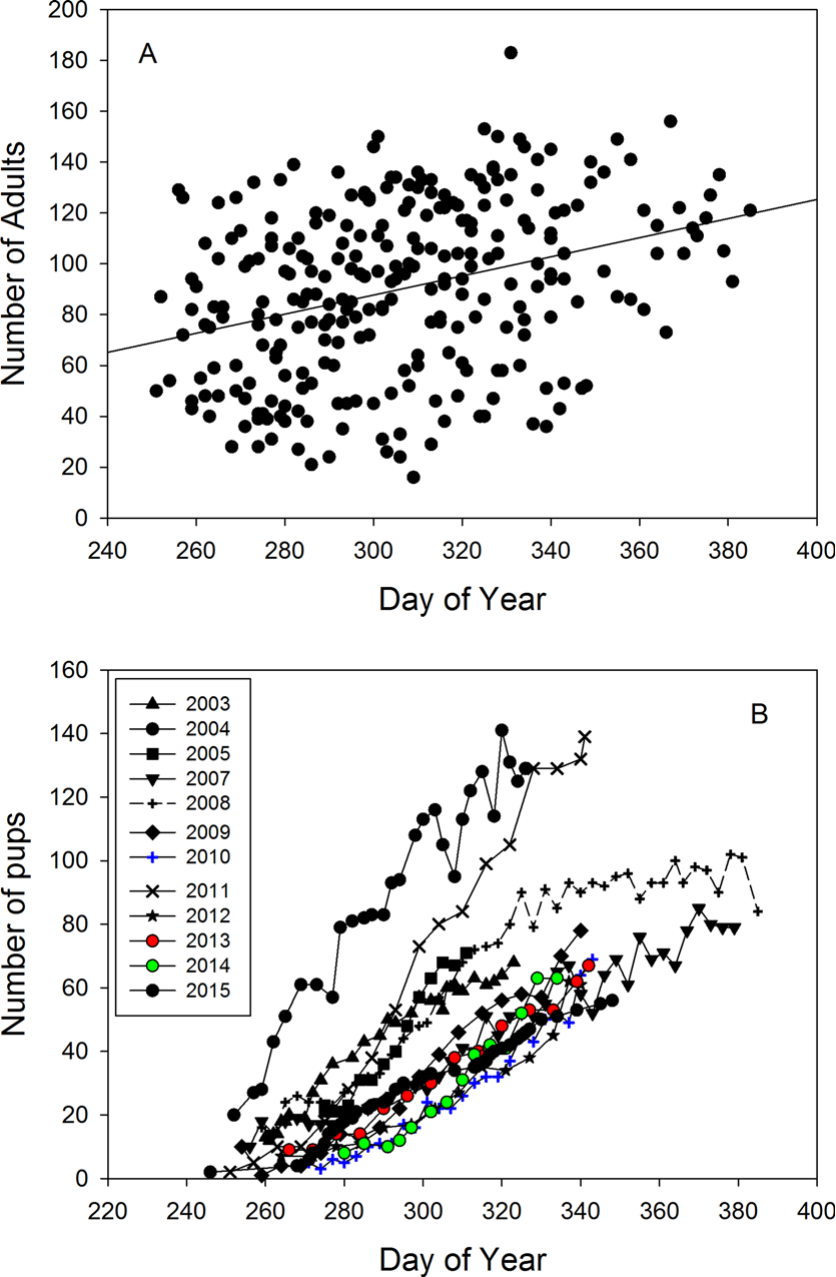
(A) Trend in adult numbers ashore with date (years 2003–2015, without 2006; Jan 1^st^ = day 1) (R^2^ = 0.158, t = 6.93, p < 0.0001) correlates (r = 0.647, p < 0.001) with (B) the increase in pup numbers with date. Day 240 = 29^th^ of August. In 2007 and 2008 census work extended into January (here graphed as days 366–390) showing the levelling off of pup counts at the end of the reproductive season. No pup census data for 2006.

Date (as day of the year counted from January 1^st^ as 1) was a significant predictor of pup numbers as pup numbers increase over the reproductive season. Accordingly, for all years, pup numbers counted were highly correlated with date (R^2^ = 0.45, F_1,249_ = 208.8, p < 0.0001). As a consequence, in all mixed effects models including the highly collinear variables pup number and date, date becomes insignificant. Therefore, we calculated models (except for pup numbers counted) using the number of pups in a given census as the predictor variable rather than date. We decided on this modelling approach, since the number of pups born at a certain date varied greatly among years (Figs 2 and 3B), thereby differentially influencing female presence and likely indirectly also male attendance at the colony. The increase of adult animals counted across the cold-R season correlates strongly with the number of pups counted (Fig 3A and 3B; LME estimate: 0.49, t = 11.58, p < 0.0001). Daytime significantly influenced the numbers of adults counted. More adults (irrespective of sex) were found ashore during the cooler morning and afternoon periods of the day than around mid-day (LME estimate for adults of both sexes: -8.17, t = -2.74, p = 0.003), but this did not apply to immature animals (LMM estimate: 1.29, t = 0.6, p = 0.55). Short term fluctuations in SST as reflected by SST_10_ influenced only female numbers with fewer females present at higher SST_10_ (LME estimate: -4.80, t = -3.54, p = 0.002). The number of females counted was also lower around mid-day (LMM estimate: mid-day -5.19, t = -2.44, p < 0.01).

Male numbers were similarly affected by total pup numbers (counted during a given census) and daytime, with lowest numbers counted around mid-day (LME pups: estimate 0.13, t = 8.82, p < 0.001; mid-day: estimate -2.80, t = -2.59, p = 0.005).

Immature numbers varied among years as a consequence of the variance in numbers of pups born in the previous year. Immature numbers encountered in a cold-R season census were only influenced negatively by pup number (LME estimate: -0.13, t = -4.05, p < 0.001). As Fig 2 shows, the total number of pups born (determined from monitoring all parturitions during the respective study periods) fluctuated substantially among years. As suggested by the simple correlation reported above, the mixed effects model also demonstrated that pup numbers increased with date (LME estimate: 0.86, t = 34.01, p < 0.0001) and in addition there was a weak trend towards higher numbers being counted at high tide (LME estimate: 2.50, t = 1.52, p = 0.065), most likely because pups are more difficult to detect along wide rocky tidal shores.

We also tested the effects of environmental parameters on the cold-R season census data of individual years (2003–2005, 2007–2015) using linear models. In all years, pup number was significantly influenced by date (always p < 0.001; Fig 3B). In eight out of the 12 years (except in 2003 and 2012–2014), pup number was a significant predictor of the number of adults counted (p < 0.01, adjusted R^2^ > 0.23). Categorical daytime and tide level at the beginning of the census had no influence on adult numbers counted. SST_10_ significantly predicted adult numbers ashore in only 4 out of 12 years (2005, 2007, 2009, 2015), with higher values predicting more animals ashore. More immatures were counted in 2003 at high tide (LM estimate: 10.15, t = 2.26, p = 0.03) and in the morning and around mid-day than in the evening (LM estimate: 7.7, t = 2.81, p = 0.009). No influence of tide level and time of day on immature numbers was found for the other individual years.

#### Warm-nR season

With fewer years to analyze and the lower number of counts, results for the warm-nR season are less clear. SST_10_ had no influence on the number of animals counted ashore in the warm-NR season (n.s.). More adults were found ashore during the cooler morning and late afternoon periods of the day than around mid-day (LME estimate mid-day:-27.06, t = -4.26, p < 0.001). Numbers were also sensitive to date effects: The later in the warm-nR season we conducted a census, the fewer adult animals were seen ashore (LME estimate: -0.67, t = -3.06, p < 0.003). The number of juveniles counted (including moulted and unmoulted pups of the previous breeding season as well as immatures) was also affected by time of the day, with fewer juveniles counted around mid-day (LME estimate mid-day: -10.81, t = -2.32, p = 0.02) and more at high tide (LME estimate 8.84, t = 2.01, p = 0.047).

We have sufficient data to estimate seasonal effects within a given year only for 2003. In that year, only the date effect on adult numbers was significant (LM estimate: -0.973, t = -3.49, p = 0.0014).

### Overall trend in population size

We assessed a potential change in population size **among** years, taking into account those factors which we found to have a significant influence on census numbers within seasons.

#### Cold-R season

**Fig 4:**
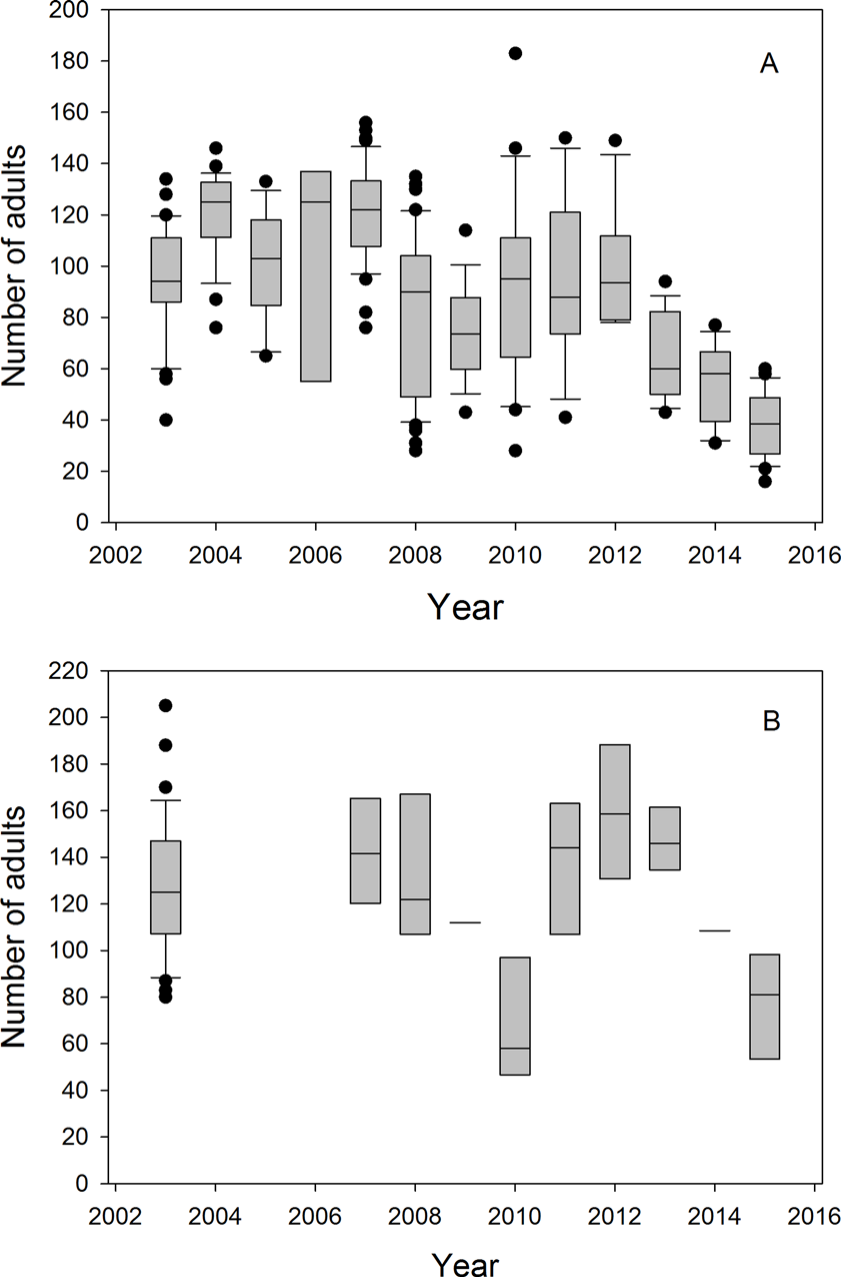
(A) Year to year cold-reproductive season variability in total number of adults counted ashore (only four census dates for the year 2006). Boxes give medians and quartile ranges, barbs 10–90% ranges, dots indicate outliers. (B) The same for warm-non-reproductive seasons, for which data are available.

For the total number of all animals (excluding pups) as well as for the sub-categories (all adults, adult females, adult males, immatures and pups), ‘Year’ negatively influenced the numbers counted (Table 2), suggesting a decline in numbers over the last 13 years (Fig 4A). However, there was substantial variability among years in the number of adults ashore (Fig 4A). This appeared to be influenced by SST_C_ (the mean SST averaged over September to December) as suggested, for example, by the lower numbers of adult in the warm years 2008 and 2009 compared to 2007 (Fig 1 and Fig 4A). In general, adult and immature numbers were negatively influenced by SST_C_ (Table 2) probably due to large differences in marine productivity and consequent changes in absence duration (see below).

**Table 2:** Best linear models for census numbers as a function of year (2003–2015) for the cold-reproductive season.

modeled variable	intercept	Year	SST_C_	number of pups	daytime (mid-day)	F, df	Multiple R^2^ adjusted
total adults	5640 p<0.0001	-2.634 p<0.0001	-12.548 p<0.0001	0.467 p<0.0001	-8.713 p = 0.0053	92.43 5, 267	0.63
adult females	3235 p<0.0001	-1.484 p<0.0001	-9.230 p = 0.0001	0.338 p<0.0001	n.s.	115.1 3, 269	0.56
adult males	1446 p<0.0001	-0.678 p<0.0001	-3.002 p<0.0001	0.113 p<0.0001	-3.699 p = 0.0013	41.17 5, 267	0.42
immatures	6083 p<0.0001	-2.876 p<0.0001	-9.659 p<0.0001	-0.185 p<0.0001	n.s.	53.69 3, 269	0.37
pups	9140 p<0.0001	-4.682 p<0.0001	3.380 p = 0.0023	**date** 0.771 p<0.0001	n.s.	151.37 3, 269	0.62

Non-significant factors were removed in the final model. SST_c_ is the mean sea surface temperature (SST) for September to December of the respective year. Day of year is calculated from January 1^st^ as day 1 of the year. Pup number is the number of pups counted at the date of the respective census. All models are significant at p<0.0001.

#### Warm-nR season

Models for the warm-nR season have less support than those for the cold-R season. As moulted young born in the previous birth season are often not reliably distinguished from small yearlings, we combined the categories of immatures and pups and, for analysis of warm season data, together called them ‘juveniles’. Year, date, and time of day influenced the number of adults counted. Overall, the number of adults decreased during the warm-nR season over the last 13 years (Fig 4B; Table 3). SST_W_ had no demonstrable effect on the number of animals ashore.

**Table 3:** Best linear models for census numbers as a function of year (2003–2015) for the warm-non-reproductive season.

modeled variable	intercept	Year	SST_w_	date	daytime (mid-day)	F df	Multiple R^2^
total adults	5790 p<0.0015	-2.778 p = 0.002.	n.s.	-0.867 p<0.0012	-32.342 p<0.001	12.41 4, 77	0.36
adult females	-2001 p = 0.0801	1.047 p = 0.0648	n.s.	-0.390 p = 0.0044	-19.178 p = 0.0002	12.95 4, 77	0.37
adult males	42.2 p<0.0001	n.s.	n.s.	n.s.	-13.189 p = 0.0004	9.09 2, 79	0.17
Juveniles (Immatures and pups)	-1201 p<0.0001	6.198 p<0.0001	-11.47 p = 0.055	-0.562 p = 0.0179	-14.24 p = 0.089	13.8 5, 76	0.44

Non-significant factors were removed in the final model. SST_w_ is the mean sea surface temperature (SST) from January to April. Day of year is calculated from January 1^st^ as day 1 of the year. Pup number is the number of pups counted at the date of the respective census. All models are significant at p<0.0001. Data for 2003 were modelled using a reduced data set with only 9, roughly equally-spaced censuses to avoid excessively weighting that season.

### Estimating colony size from census numbers

#### Cold-R season

**Fig 5:**
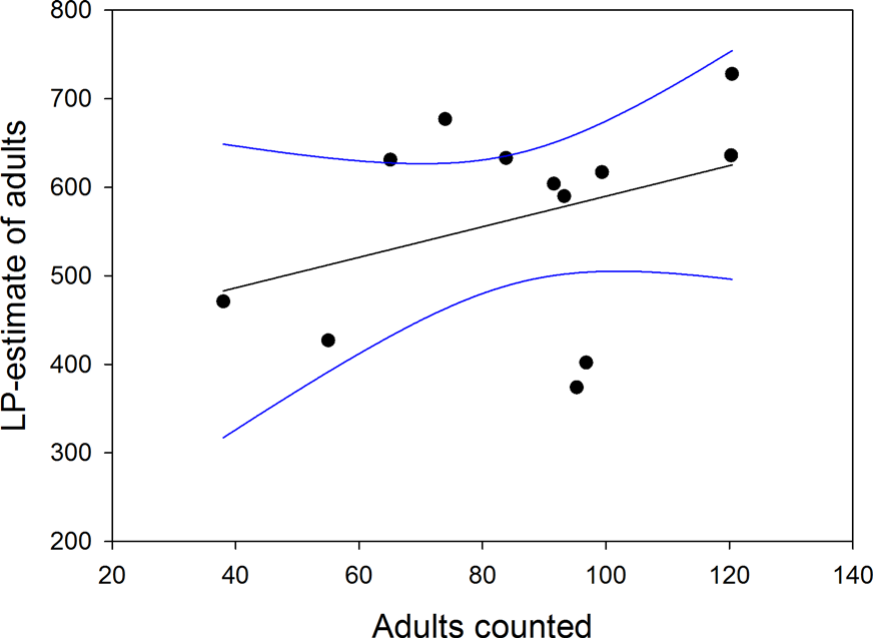
Lincoln-Petersen (LP) estimates (yearly means) of adult numbers in the cold season were uncorrelated with yearly mean numbers of adults counted. LP estimate = 417.4 + 1.725 * census mean (F_1,10_ = 1.54, p = 0.243). The lines indicate 95% confidence intervals. No estimate for 2006.

Tables 4 and 5 show the mean Lincoln-Petersen (LP) estimates of adults and immatures per year for the cold-R seasons. The overall mean of estimates was 565 ± 116.3 adults of which we counted 16.04 ± 5.6% in a given census. Across the study years, we observed between 8.3% and 24.3% (27.5% in 2003) of the total estimated number of adults in our censuses. In 2003, the number of marked adults was low and almost doubled during the observation period. Therefore, the 2003 as well as the 2005 estimate, when we only stayed for a short time in the colony, need to be interpreted with caution. LP estimates showed no clear trend in numbers across years (adults = 1.5 * year -2951; adjusted R^2^ = -0.096, df = 10, p = 0.854). Yearly means of LP estimates of adults were not correlated with yearly means of the raw census values (Fig 5; adjusted R^2^ = 0.046, df = 10, p = 0.244), pointing to a strong influence of changes in attendance patterns across years (compare Table 4). However, this result needs to be interpreted with caution as the power of the test (0.209) is low.

**Table 4:** Lincoln-Petersen (LP) estimates of the total number of adults for the cold-R seasons of the years 2003 to 2015.

Year	Mean LP estimate	CI	Prop. counted	Mean counted	Total tagged
**2003**	544	378–710	0.207	95	155
**2004**	636	490–782	0.192	120	216
**2005**	402	299–505	0.243	97	123
**2006**	--	--	--	--	--
**2007**	728	614–842	0.168	120	381
**2008**	633	514–752	0.133	84	342
**2009**	677	543–811	0.110	74	377
**2010**	604	503–705	0.156	92	364
**2011**	590	490–690	0.164	93	348
**2012**	617	536–698	0.163	99	408
**2013**	631	490–772	0.106	65	322
**2014**	427	340–514	0.132	55	251
**2015**	471	321–621	0.083	38	207
**All years**	**580**	**460–700**	**0.155**	**86**	**291**

For the cold-R season of 2006 data by category are too few to be usefully evaluated. Prop. counted: The mean for all censuses in the cold-R season of a given year of the proportion of animals of the respective category that was actually seen during a given census. Mean counted: The mean of adult animals recorded in individual censuses. Total tagged: Total number of tagged individuals seen during the entire respective season.

**Table 5:** Lincoln-Petersen (LP) estimates of the total number of immatures (without pups) for the cold-R seasons of the years 2003 to 2015.

Year	Mean LP estimate	CI	Prop. counted	Mean counted	Total tagged
**2003**	227	207–247	0.388	86.5	122
**2004**	231	220–242	0.385	88.7	172
**2005**	239	232–247	0.455	108.1	221
**2006**	--	--	--	--	--
**2007**	290	285–295	0.377	109.6	288
**2008**	252	248–256	0.235	59.3	250
**2009**	240	234–246	0.346	83.3	227
**2010**	204	200–208	0.332	67.4	199
**2011**	220	218–222	0.308	67.6	219
**2012**	302	297–307	0.399	120.9	294
**2013**	251	245–257	0.378	95.2	235
**2014**	155	151–159	0.349	53.7	148
**2015**	146	134–158	0.283	41.1	135
**All years**	**230**	**223–237**	**0.353**	**81.8**	**209**

For the cold-R season of 2006 data by category are too few to be usefully evaluated. Prop. counted: The mean for all censuses in the cold-R season of a given year of the proportion of animals of the respective category that was actually seen during a given census. Mean counted: The mean of adult animals recorded in individual censuses. Total tagged: Total number of tagged individuals seen during the entire respective season.

Most immatures were marked, but marked 3 and 4 year olds were observed only rarely during censuses. This led to close correspondence between the number of marked animals and LP estimates of immatures (Table 5). Overall, we estimated 230 immatures in the population of which only 35.3% were observed in a given census. There was no clear trend in immature number estimates over time (immatures = -4.582*year + 9436; r = 0.265, p = 0.206). Pup numbers counted reflect the number of alive pups very well (more than 90% of existing pups are usually counted; data not shown), but total numbers born varied greatly (Figs 2 and 3B) without a clear trend across years (r = 0.144; F_1,11_ = 0.233, p = 0.639; N = 13 years).

#### Warm-nR season

More adults were counted in a given census during the warm-nR compared to the cold-R season. Again, numbers varied substantially from year to year (Fig 4B). The overall mean estimate of adult numbers for the warm-nR seasons of 2003, 2007, 2008, 2011–2015 was 568 individuals, of which we could observe about 23% in a given census (Table 6). The proportion of adults and juveniles seen was generally substantially higher during the warm-nR than during the cold-R season (Tables 6 and 7 versus Tables 4 and 5). There was no discernible trend in numbers across years (R^2^ = -0.074, p = 0.555) and also no correlation between directly counted numbers of adults and numbers estimated by LP estimates (R^2^ = -0.083, p = 0.592).

**Table 6:** Lincoln-Petersen (LP) estimates of the total number of adults for the warm-nR seasons of the years 2003, 2007–2015.

Year	Mean LP estimate	CI	Prop counted	Mean counted	Total tagged
**2003**	640	395–885	0.217	127.6	94
**2007**	651	528–774	0.216	142.5	239
**2008**	377	323–431	0.361	134.0	179
*2009*	*460*	*381–539*	*0*.*244*	*112*.*0*	*207*
**2010**	647	507–787	0.111	67.3	300
**2011**	499	424–574	0.281	140.7	229
**2012**	629	550–708	0.259	160.2	339
**2013**	580	480–680	0.261	149.0	225
*2014*	*418*	*354–482*	*0*.*259*	*108*.*5*	*216*
**2015**	517	390–644	0.157	78.5	206
**All years**	**568**	**450–685**	**0.233**	**125.0**	**226**

Based on the total number of marked animals of the respective category actually seen during the respective season (Total tagged). 2009 and 2014 values (in italics) are based on 2 counts only. These two years were not included in the calculation of the overall means. CI = 95% confidence interval of the Lincoln-Petersen estimate. Prop. counted: The mean for all censuses of a given year of the proportion of animals actually seen during a given census. Mean counted: The mean of animals recorded in individual censuses.

**Table 7:** Lincoln-Petersen (LP) estimates of the total number of juveniles (pups plus immatures) for the warm-nR seasons of the years 2003, 2007–2015.

**2003**	286	234–338	0.425	118.9	122
**2007**	339	329–349	0.548	185.5	328
**2008**	301	295–307	0.607	180.2	313
*2009*	*163*	*151–175*	*0*.*671*	*109*.*5*	*122*
**2010**	279	248–310	0.426	113.3	206
**2011**	247	233–261	0.507	124.6	229
**2012**	331	315–347	0.511	169.8	302
**2013**	391	364–418	0.498	180.3	302
*2014*	*228*	*218–238*	*0*.*642*	*144*	*204*
**2015**	251	234–268	0.446	108.2	215
**All years**	**303**	**282–324**	**0.496**	**147.6**	**252**

Based on the total number of marked animals of the respective category actually seen during the respective season (Total tagged). 2009 and 2014 values (in italics) are based on 2 counts only. These two years were not included in the calculation of the overall means. CI = 95% confidence interval of the Lincoln-Petersen estimate. Prop. counted: The mean for all censuses of a given year of the proportion of animals actually seen during a given census. Mean counted: The mean of animals recorded in individual censuses.

## Discussion

Our data revealed an enormous variation in the number of animals counted ashore, both within and between seasons. We analyzed the number of counted adults and immatures to understand which factors influence census data and thus may affect population estimates and trends. Time of day and day of season (correlated with the number of pups counted in the cold season) explained part of the variance in adult numbers within a given season. Among years, adult numbers increased with lower SST_c_ in the cold season, but interestingly the highest numbers were observed in the warm season. Raw counts suggested a slight decline in numbers in the cold, but not in the warm season. The fact that LP estimates indicated no change in the cold season suggests that a change in individuals’ attendance patterns rather than a change in population size explains the apparent trend in adult numbers. Our best estimate of population size is about 810 individuals, excluding pups, in the cold season and 871 (including pups of the previous season) in the warm season. Our estimate thus agrees quite well with the previous estimate by [19] of 875 (CI 791–958; including about 80 pups) for the cold and 970 (CI 848–1,143) for the warm season. Nevertheless, our 13 year time series of census data continues to produce wide confidence intervals for population size and does not produce a simple, clear answer about population trends [10].

### Changes in numbers across the reproductive and non-reproductive season

On average, only about 16% of the adults and about 35% of the immatures are counted in a given cold-R season census. The low observability results from the fact that adults and older immatures spend much time at sea foraging [16,34,35] and younger animals often swim close to shore where many escape detection. However, conducting counts in the hope to obtain reliable population estimates is further complicated as considerable variability exists in the number of animals encountered ashore. Counts during the morning and evening resulted in the largest numbers of animals observed. Avoiding land during the hottest time of the day allows the animals to minimize thermoregulatory problems.

The date also affected census counts. Counts later in the warm season resulted in lower numbers of adults and immatures, presumably related to the increase in SST with date. However, if SST were entirely responsible for this effect, we would expect SST_10_ to influence census results and we should observe similar trends during the cold-R season. Neither of these effects was found. In contrast, during the cold-R season, adult numbers increased with date (as did SST). These increases in adult numbers with progressing date were more strongly related to the number of pups already born than to date and SST_10_. As females with newborn young tend to spend more time ashore and make shorter trips to sea [35] than females with older young or without dependent offspring, this will increase the number of adults ashore. Simultaneously, the presence of more females may attract males [29,36], who appear able to assess female densities [37]. Still, adult and immature numbers during the cold-R season were negatively influenced by SST_c_ (the mean SST averaged over September to December), probably due to large differences in marine productivity, prey distribution [38] and consequent changes in absence duration.

Why adults are more likely to be seen ashore during the warm-NR season than during the cold-R season remains unclear. Part of the answer may lie in the observation that a greater number of male individuals come ashore or that individual males spend more time ashore. This change may be related to lower competition among males for access to females in the non-breeding season [36]. However, the same trend was also observed for females and this remains unexplained at present. Most likely, other variables like food abundance and position of food patches (distance from the island as well as depth distribution of prey) relative to the study island influence the number of animals encountered ashore. Our results show that census numbers across seasons seem to be related to environmental influences but, during the cold season, also relate to the number of pups born in a given season. As this number varies tremendously from year to year, so does the number of adults counted ashore.

### Trends in population size or fluctuations due to environmental conditions?

Our results allow no firm conclusions about a trend in population size yet. Raw counts suggested a decline in numbers. Whether this decline depends on the El Niño-like effects of warmer periods like 2008, 2009 and 2015 or signals a real decline in numbers is difficult to judge. Interestingly, the raw numbers and the LP estimates did not directly correlate with each other and LP estimates indicated no trend. This discrepancy most likely relates to differences in attendance of adults in the colony which varied strongly across study years between 8% and 24% in the cold and 11% and 36% in the warm season. As an example, take the number of adults counted in 2003 and 2014: in the former year, we counted on average 95 adult individuals, but only 55 in 2014. However, these numbers largely reflect differences in absence duration, as we observed 21% of adults ashore in 2003, versus only 13% in 2014. Additionally, LP estimates vary substantially from year to year, for example resulting in 544 adults for 2003, 636 in 2004 and 631 in 2013 versus only 427 in 2014. This variability from year to year appears to be related to environmental influences but, during the cold season, also relates to the number of pups born in a given season, which varied strongly among years. At our colony, females lactate their offspring approximately for 2 years [39]. If females are not as likely to support a pregnancy successfully while suckling an offspring than when without a dependent offspring, this could influence pup numbers born in the following year and partly explain the approximately 2-year cycle of pup numbers observed in the majority of years. In conclusion, even a monitoring period of 13 years did not result in a simple, straightforward answer with respect to the population trend, given the large fluctuations in reproduction and presence/ absence of animals from the colony.

### Assumptions and caveats

As explained above in the Method section, our estimates assume that all the animals we see in the colony actually belong to it, i.e. the population is closed. This seems to be true for the immatures and juveniles given that our LP estimates are so close to the actual numbers of tagged animals (see also [32]). Indeed in the later years of the study we had essentially all juveniles marked, at least late in the season. For adults, we know that they show very high site fidelity [19,29]. However, we also know that during their foraging sojourns, adult females haul out at places other than their home colony [15,16]. This could lead to the inclusion of animals from other colonies in our censuses and might cause errors, which we presently cannot account for. We also have strong hints that 3 and 4 year-olds are wide ranging and only rarely visit the home colony which means that our population is not as closed as we implicitly assume in our estimation procedure. Whether these potential biases lead to over- or underestimation of numbers of individuals belonging to the study colony is unclear. However, the fact that marked animals from our colony are seen very rarely at other, close-by colonies suggests that our assumptions are largely met. The question can best be addressed by modelling the data using a model for an open population, which requires detailed consideration of the fate of all marked individuals, tag losses and may suffer from biases due to the different length of our observation periods in different years.

### Recommendations

As our results demonstrate, the interpretation of census data is far from straightforward. The attendance of animals varies substantially within and among years, thereby diminishing the value of single counts in individual years in terms of monitoring the population’s status. These findings likely also apply to other species of seals.

For the Galapagos sea lion, we recommend that censuses should be done at the same time of day, preferably in the morning or late afternoon, to ensure a maximum number of animals ashore. Tide level and SST have little influence on numbers found ashore. However, extreme SSTs during El Niño or la Niña events may show much stronger influence, given that the animals tend to stay away for much longer periods during El Niño [23,24] and the opposite may be true during La Niña episodes. As pup numbers increase during every breeding season and we here show that this influences the number of adults ashore, the greatest numbers of adults are counted late in the season, which on Caamaño and other islands in the center of the archipelago, is towards the end of December. In areas where marine productivity and SSTs are different from conditions in the central archipelago, best census times may differ. This could apply to the colder, more productive western area (western Isabela and Fernandina), and the northern, more tropical islands (Pinta, Marchena and Genovesa). At all sites, a census towards the end of the reproductive period would produce a reasonable estimate of the number of pups produced by a local population, thereby providing another useful index of population size. However, given the major variability in pup numbers across years, monitoring should definitely continue over many years, and preferably be based on repeated counts over at least one week since a single point estimate appears unreliable. Given that the population trend of the Galapagos sea lion is unclear and that this highly iconic species has recently been classified as endangered by the IUCN [18], our study highlights the need for continued monitoring preferably over a wider area within the archipelago.

## Supporting Information

S1 Table Census_warm_cold. (XLSX)

S2 Table Data used for LP estimates. (XLSX)
